# Mediating Role of Psychological Maladjustment in Relation Between Dark Triad, Psychological Distress and Subjective Happiness of Pakistani Emerging Adults

**DOI:** 10.3389/fpsyg.2022.906334

**Published:** 2022-07-08

**Authors:** Asia Mushtaq, Ayesha Inam, Arooj Najmussaqib, Anjum Afshan, Eda Ermagan-Caglar

**Affiliations:** ^1^Department of Applied Psychology, National University of Modern Languages, Islamabad, Pakistan; ^2^Department of Humanities, COMSATS University, Islamabad, Pakistan; ^3^Bilquis Postgraduate College for Women, Rawalpindi, Pakistan; ^4^Affiliated College of Air University, Islamabad, Pakistan; ^5^Department of Psychology, Beykoz University, Istanbul, Turkey

**Keywords:** Dark Triad, psychological maladjustment, subjective happiness, psychological distress, emerging adults

## Abstract

The transition from adolescence to adulthood is fraught with challenges that might have impacts on later life and personality development. Earlier research investigated Dark Triad traits in connection to emotional problems. The current study, on the other hand, focused on investigating the mediating role of psychological maladjustment in the relation of Dark Triad traits, psychological distress, and subjective happiness in emerging adults. A sample of 546 participants aged 18–25 years (M = 21.2 years) from Pakistan have participated to complete an online survey. Standardized assessment tools were used to measure the targeted variables. Results indicated that Machiavellianism and psychopathy were positively associated with psychological distress, whereas narcissism appeared to be a non-significant predictor. Subjective happiness was positively associated with Machiavellianism and negatively associated with psychopathy. In addition, mediation analysis through Structural Equation Modeling (SEM) indicated that the Dark Triad traits (Machiavellianism and psychopathology), psychological distress, and subjective wellbeing were explained by psychological maladjustment. Implications and limitations are discussed.

## Introduction

The Dark Triad (DT) consists of three overlappings, yet distinctive personality traits: psychopathy, narcissism, and Machiavellianism (Paulhus and Williams, 2002; Jonason and Krause, 2013). These traits share a core of manipulation, callousness, grandiosity, and selfishness (Jones and Figueredo, 2013). Hence, all three of these traits are regarded as disrespect for societal norms, which often leads to social indiscretions such as lying, cheating, manipulating, and stealing. Furthermore, possessing dark characteristics is associated with dysfunctional interpersonal relationships due to self-centered behavior and a lack of concern for others. It should be noted that the DT traits are considered subclinical traits and are not concerned with categorical disorder labeling or clinical disorder (Lyons, 2019). Narcissism is characterized by having an inflated, grandiose, and, often, unrealistic sense of self (Raskin and Terry, 1988; Giacomin and Jordan, 2016).

Narcissists have low empathy for others resulting in manipulating and exploiting relations for their achievements and acknowledgments (Campbell et al., 2011). Narcissistic individuals are typically egotistic, dominant, self-centered, and self-entitled (Sabouri et al., 2016). Machiavellianism is characterized by immoral flattery, deceit, emotional manipulation, and dishonesty (Jones and Paulhus, 2009). Machiavellian individuals are typically described as being callous, manipulative, and more strategic than impulsive (Christie and Geis, 1970; Jonason and Krause, 2013). In contrast, individuals with psychopathic traits display limited self-control, and impulsive and adventurous behavior (Del Gaizo and Falkenbach, 2008; Sabouri et al., 2016). Studies suggested that Machiavellianism is related to psychopathy (Egan et al., 2014; Vize et al., 2018; Lyons, 2019; Rogoza and Cieciuch, 2020) and a meta-analysis of 91 studies showed that Machiavellianism and psychopathy were highly correlated with each other (Muris et al., 2017). Finally, psychopathy is most likely the “darkest” of the Dark Triad characteristics. It is distinguished by selfishness, deception, and lack of sympathy (Levenson et al., 1995). According to the triarchic model, psychopathy comprises boldness, meanness, and disinhibition (Patrick et al., 2009). In comparison to the other two traits, psychopaths are known to have more disregard for others, manifested in disruptive interpersonal behaviors such as bullying (Baughman et al., 2012), partner abuse, and sadism (Carton and Egan, 2017).

## Relation Between Dark Triad Traits, Psychological Distress, And Happiness

Emerging adulthood (ages 18–25 years) refers to the transitional period when individuals leave late adolescence and enter adulthood (Arnett, 2000). This phase is frequently defined by significant personal and life changes, such as identity development (Waterman, 1982), attending college/university, becoming independent and making key life decisions, choosing a career and commencing full-time employment, financial independence, marriage, and probably parenting (Barlett and Barlett, 2015). Although Pakistan is a collectivistic society, family is given importance and even adolescents, young adults, and adults have lesser autonomy for taking decisions related to the choice of life partner, career, etc. The average age of earning for educated youth starts after 25 and may extend up to 30 years depending on the availability of jobs. However, contribution to household earnings does not necessarily mean financial independence because of the family structure in Pakistan where adults live with their parents despite being financially independent and, the eldest, usually the father, is considered the head of the family (Khawar and Sarwar, 2021). In a collectivistic society, Pakistani parents usually apply authoritarian parenting styles (i.e., harsh and bossy) and literature clearly linked the (non-authoritative) parenting styles with DT traits in parents and their offspring (Geher et al., 2020). Within the past few decades, globalization has created a tremendous impact on the lives of people and gradually changed the familial relationships, family structure and norms, gender roles, identity, work, and women's and children's rights in collectivistic cultures too (Yang and Neal, 2006). With this global transition, Pakistani youth also demand autonomy, independence, and family norms and relationship are changing. Single unit (non-extended) familial structure becoming popular in Pakistan.

Because of crucial life challenges, emerging adults experience high levels of mental health concerns and most clinical disorders emerge during adolescence and young adulthood (The Centre for Addiction Mental Health CAMH, 2020). In a recent meta-analysis, Khan et al. (2021) reported a 42.66% prevalence of depressive symptoms among university students in Pakistan. As emerging adults transition into their new identities and attempt to navigate through their social world full of life changes, Dark Triad traits are likely to develop (Klimstra et al., 2020). According to Barlett and Barlett (2015), Dark Triad traits are associated with emerging adulthood facets (e.g., negativity, other-focused, and feeling in-between). Identity exploration was only related to psychopathy, whereas other-focused was negatively related to all the Dark Triad traits except narcissism (Barlett and Barlett, 2015). Second, younger individuals exhibit more Dark Triad characteristics than older adults; potentially because of having lower self-control (Jonason and Tost, 2010). Additionally, self-control is adversely connected to Machiavellianism, narcissism, and psychopathy (Jonason and Tost, 2010). Dark Triad traits and low self-control (i.e., risk-taking, novelty-seeking, and impulsivity) are also considered to be risk factors for psychopathology (Hsu et al., 2012).

Jonason et al. (2015) reported high levels of depression in relation to each Dark Triad trait, however, only narcissism was related to increased anxiety. Moreover, both psychopathy and Machiavellianism have been linked with increased reports of anxiety (Pennington et al., 2015; Czibor et al., 2017) and depression (Jonason et al., 2015; O'Boyle et al., 2015; Pennington et al., 2015). Anxiety or low empathy is associated with the three DT traits (Miller et al., 2010; Jonason and Kroll, 2015; Megías et al., 2018), and difficulty in regulating mood and alexithymia associated with psychopathy and Machiavellianism (Cairncross et al., 2013; Love and Holder, 2014; Miao et al., 2019).

Happiness is an emotional state characterized by feelings of joy, cheerfulness, satisfaction, and serenity. Happiness has many definitions, usually associated with positive emotions, feelings, and life satisfaction (Diener et al., 1999; Diener, 2000, 2021); happy individuals are more likely to be flourishing people (Lyubomirsky et al., 2005). Within the dark triad research, previous literature is heavily loaded with inquiries related to subjective wellbeing (Joshanloo, 2021). The relation between DT traits and happiness is limited either no association was found between these variables (Aghababaei et al., 2014; Aghababaei and Błachnio, 2015), or Machiavellianism and psychopathy are related to lower positive mood (Egan et al., 2014). Similarly, some other studies reported a non-significant correlation of Machiavellianism and psychopathy with happiness, and positive associations of narcissism with the orientations to happiness (Pollock et al., 2016; Limone et al., 2020). The Dark Triad traits are also linked to happiness in terms of attaining status, power, goals, social bonding, and mate partner selection (Jonason and Tome, 2019).

## Psychological Maladjustment As A Mediator

Maladjustment is usually considered an individual's limitation in response and emotional reactions that can be grouped as undesirable personality characteristics and a negative personality pattern (Kuppens et al., 2010). The regulation and control of emotions are also severely hampered for these individuals. One of the major tasks of emerging adulthood is establishing intimate relationships (Erikson, 1982). According to the DSM-5 alternative personality disorder model, antagonism consists of several characteristics including manipulativeness, grandiosity, attention-seeking, hostility, callousness, and dishonesty. Antagonism is found to be a common feature of all three Dark Triad traits. Individuals with high-end Dark Triad traits are known to have toxic interpersonal relationships and often show hostility toward other people (Grigoras and Wille, 2017). Due to a lack of empathetic nature, their relations including spouses, family members, and friends suffer more than the individual themselves (particularly in Narcissism) (Lyons, 2019). Furthermore, Grigoras and Wille (2017) showed that hostility was a strong predictor of Machiavellianism and narcissism. Narcissism was associated with reduced negative affect and decreased detachment, indicating that narcissism is also related to experiencing positive emotions and a want to be in contact with people, i.e., dependence on obtaining attention. Psychopathy was associated with disinhibition and could lead to impulsive and maladaptive behaviors.

Several studies highlighted that all Dark Triad traits are linked with low agreeableness, indicating that individuals high in the DT traits are unfriendly, cold, and argumentative (Paulhus and Williams, 2002; Jakobwitz and Egan, 2006). However, a meta-analysis of 91 studies found that all three traits were negatively correlated with agreeableness, whereas psychopathy and Machiavellianism had a stronger correlation than narcissism (Muris et al., 2017). Among other traits, vindictiveness and coldness are two of the Dark Triad's most prominent characteristics. Individuals with a high level of the Dark Triad characteristics exhibit little concern for others and prioritize their own selves. Similarly, Petrides et al. (2011) investigated the relationship between emotional intelligence and the Dark Triad, and results showed that narcissism was correlated with higher, and Machiavellianism and psychopathy with lower self-assessed emotional intelligence. It is learned that people high on the Dark Triad have little empathy and a lack of pro-social emotions, which facilitates their exploitative character (Lyons, 2019). These assumptions are supported by the findings of Jonason and Krause (2013), who showed that psychopathy, in particular, had a negative connection with empathy.

**Figure 1:**
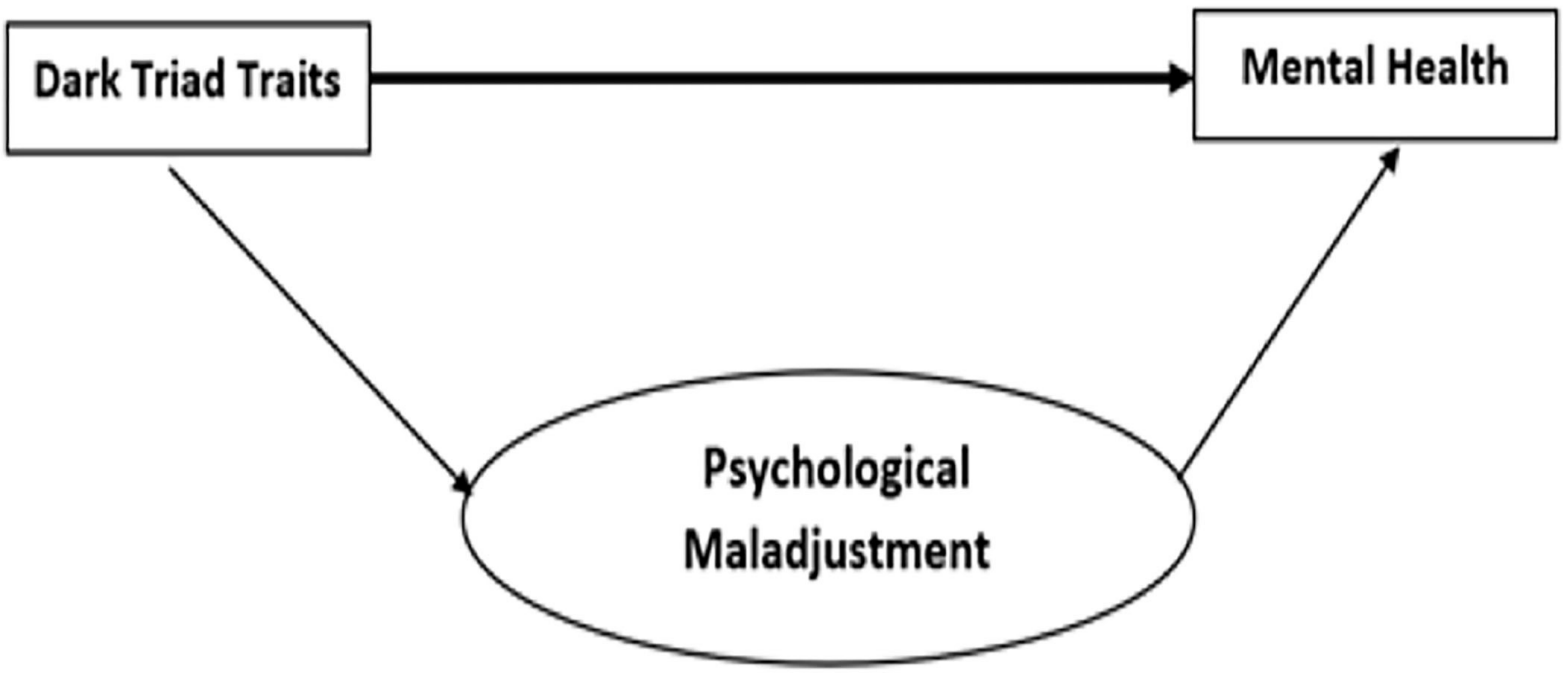
Conceptual model of the study.

Psychological maladjustment can also be grouped into the different subdomains of anger, hostility, aggression, dependence or defensive independence, negative self-esteem, negative self-adequacy, emotional instability, emotional unresponsiveness, etc. (Rohner, 2004; Khaleque, 2015). The indications of psychological maladjustment include pervasive sadness over longer periods of life (Kessler et al., 2003). This also includes signs of decreased mental health and emotional soundness (Kuppens et al., 2010). In relation to behavioral concerns, a recent study demonstrated that Machiavellianism and psychopathy relate to unreliability, disorganization, carelessness, and coldness, whereas narcissism is characterized by striving to be with other people and enjoying new experiences (Vize et al., 2018). Furthermore, Stenason and Vernon (2016) observed that psychopathy and narcissism are associated with a greater prevalence of risky substance abuse than Machiavellianism. Psychopathy also has a positive relation with risky health behaviors (i.e., drinking, smoking, and drug use). Considering these interrelationships, it is plausible that the relationship between psychological distress, subjective happiness, and DT traits may be mediated by psychological maladjustment in emerging adults (see Figure 1). However, to date, this proposition has not been explored.

Considering the above-mentioned arguments, we postulate the following:

**H1:** Dark Triad traits (narcissism, Machiavellianism, and Psychopathy) are positively related to psychological distress.

**H1a:** *Narcissism is significantly and positively related to psychological distress*.

**H1b:** *Machiavellianism is significantly and positively related to*.

**H1c:** *Psychopathy is significantly and positively related to psychological distress*.

**H2:** Dark Triad traits (narcissism, Machiavellianism, and Psychopathy) are negatively related to subjective happiness.

**H2a:** *Narcissism is significantly and negatively related to subjective happiness*.

**H2b:** *Machiavellianism is significantly and negatively related to subjective happiness*.

**H2c:** *Psychopathy is significantly and negatively related to subjective happiness*.

**H3:** Psychological maladjustment mediates the relationship between Dark Triad traits (narcissism, Machiavellianism and Psychopathy) and psychological distress.

**H3a:** *Psychological maladjustment mediates the relationship between narcissism and psychological distress*.

**H3b:** *Psychological maladjustment mediates the relationship between Machiavellianism and psychological distress*.

**H3c:** *Psychological maladjustment mediates the relationship between psychopathy and psychological distress*.

**H4:** Psychological maladjustment mediates the relationship between Dark Triad traits (narcissism, Machiavellianism and Psychopathy) and subjective happiness.

**H4a:** *Psychological maladjustment mediates the relationship between narcissism and subjective happiness*.

**H4b:** *Psychological maladjustment mediates the relationship between Machiavellianism and subjective happiness*.

**H4c:** *Psychological maladjustment mediates the relationship between psychopathy and subjective happiness*.

## Materials and Methods

### Participants

A cross-sectional design was used to collect the data of emerging adults with an age range of 18–25 years (*M* = 20.2, *SD* = 1.95 years) from different cities of Pakistan after the approval of the Institutional Ethical Board. The sample was selected using the non-probability purposive technique. According to the Pakistan Bureau of Statistics (2017), the population of urban emerging adults (18–25 years) is ~9.75% of the total population. For calculation of sample size, we used formula: s = χ^2^NP (1-P) ÷ d2 (N1) + χ^2^P (1- P), where s = sample size, χ^2^ = value of chi-square for one degree of freedom at the desired confidence level (3.841), *N* = population size, *P* = population proportion (assumed to be 0.5) and d = degree of accuracy expressed as a proportion (Krejcie and Morgan, 1970; Cohen, 1988, 1992; Chuan, 2006). Calculation done using this formula gives us a sample size of 384 for a population size of above 20,000. The participants belonged to middle-class families, with 78% of students. 237 (43.41%) lived in an extended family system and the majority (96%) were unmarried (single). Due to COVID-19 third wave, there was a lockdown and closure of educational institutions in Pakistan, therefore, online mode of classrooms and WhatsApp groups were approached to collect the data from 12 March to 25 April 2021. A total of 546 participants (45.8% women) completed the online survey on a voluntary basis (see Table 1 for details). The purpose of the study was explained with the assurance of confidentiality of personal information.

**Table 1:** Demographic characteristics.

**Variables**	***f (%)***	**Mean (SD)**
**Age of the participants**		21.2 (3.73)
**Gender**		
Male	296 (54.2)	
Female	250 (45.8)	
**Education**		
Undergrads	269 (69.7)	
Postgrads	116 (30.1)	
**Marital status**		
Unmarried	524 (95.98)	
Married	22 (4.02)	
**Familial system**		
Joint Family (extended)	238 (43.6)	
Nuclear Family (non-extended/single unit)	308 (56.4)	
**Monthly family income (in Pak. Rupee)**		150,552.12 (310,116.10)
**Birth order**		
1^st^ Born	175 (32.1)	
2^nd^ Born	143 (26.2)	
3^rd^ Born	116 (21.2)	
4^th^ and above	112 (20.5)	

*F, Frequency; %, percentage*.

## Measures

### Short Dark Triad (SD3)

The SD3 (Jones and Paulhus, 2014) is a self-report measure that consists of 27 items of Machiavellianism, psychopathy, and narcissism. Items are rated on a 5-point Likert scale ranging from 1 (strongly disagree) to 5 (strongly agree). Urdu translated and validated version of SD3 was used (Riaz, 2018; Li et al., 2020; Rogoza et al., 2020; Hussain et al., 2021) to measure Dark Triad traits in the Pakistani population and Cronbach alpha reliability of DT traits is 0.61 (Machiavellianism), 0.6 (narcissism) and 0.66 (psychopathy) in the present study.

### Adult Personality Assessment Questionnaire (PAQ; Short Form)

The Adult PAQ (Rohner and Khaleque, 2005) is a 42-item self-report questionnaire designed to assess seven personality characteristics: (a) Hostility/aggression, (b) Dependence, (c)Negative self-esteem, (d) Negative self-adequacy, (e) Emotional unresponsiveness, (f) Emotional instability, and (g) Negative worldview. Items are scored on a 4-point Likert scale ranging from (4) almost always true of me to (1) almost never true of me. Scores on the Adult PAQ (short form) ranged from 42, indicating healthy psychological adjustment, to 168, indicating severe psychological maladjustment. Munaf et al. (2012) translated PAQ and established the validity of Adult PAQ for the Pakistani population. The alpha reliability of PAQ is 0.85 in the current study.

### Kessler Psychological Distress Scale (K10)

Urdu translated version of the Kessler Psychological Distress Scale (Andrews and Slade, 2001) is a simple self-report, 10 items scale. It measures anxiety and depression on a 5-point Likert type scale ranging from (0) never to (5) always. Scores are added up with a maximum score of 50 indicating severe distress, and a minimum score of 10 indicating no distress. The validated Urdu version of K10 (Waqar et al., 2021) showed good reliability (0.9) in the current study.

### Subjective Happiness Scale

Urdu translated and validated version (Bano and Sitwat, 2017) of the Subjective Happiness Scale (originally developed by Lyubomirsky and Lepper, 1999) is a self-report, 4 items scale that assesses an individual's overall happiness as measured through self-evaluation. The 7-point Likert-type scale ranges from 1 (not a very happy person) to 7 (a very happy person). A high score indicates greater happiness. The Cronbach alpha reliability of this scale is 0.70 in this study.

### Covariate Variables

Participants' gender was controlled in data analysis.

### Data Analysis

Data were cleaned and normality tests were administered to check the biases or errors in the data. Descriptive statistics, Cronbach's reliability, and correlation analysis were computed through IBM SPSS Statistics version 23 (SPSS-23). The hypothesized models of mediation were analyzed with structural equation modeling (SEM) procedures using the Analysis of Moment Structure version 23 (AMOS-23). Categorical data were presented in frequencies and percentages, whereas, for continuous variables mean values with SDs were reported. Pearson's correlation coefficient was computed to determine the direction and strength of the relationship between all variables in the structural model. In SEM, maximum likelihood estimation was employed as a global test of the model. The goodness of fit of the models was evaluated by the chi-square (χ^2^), Root Mean Square Error of Approximation (RMSEA), Goodness of Fit Index (GFI), Tucker-Lewis Fit Index (TLI), Comparative Fit Index (CFI), Normed Fit Index (NFI) and Incremental Fit Index (IFI). RMSEA <0.1 represents an acceptable fit, whereas the GFI, TLI, CFI, NFI, and IFI values >0.9 and χ^2^/*df* <3 are considered acceptable (Hu and Bentler, 1999; Schumacker and Lomax, 2004).

## Results

The descriptive statistics for study variables were computed, and skewness and kurtosis were examined (George and Mallery, 2010; Tabachnick and *and* Fidell, 2013). Average to good alpha coefficients of all the measuring scales indicated the appropriateness of these measures to use with the Pakistani population. The age of the participants yielded a non-significant correlation with the study variables, therefore not included in further analysis. Gender was found to be a significant variable that correlated significantly with the study variables (see Table 2 for gender differences), therefore for intercorrelation by controlling the effects of gender; the partial correlation was computed (see Table 3). According to the results, narcissism is not significantly associated with the outcome variables (i.e., psychological distress and subjective happiness) whereas the other two traits (Machiavellianism and psychopathy) are strongly associated with distress and happiness. The Dark Triad traits (Machiavellianism, narcissism, and psychopathy) are highly correlated with each other. Therefore, covariance effects are also added to the structural equation model path.

**Table 2:** Gender differences in study variables (*N* = 546).

	**Male (*****n*** **=** **296)**		**Female (*****n*** **=** **250)**				**95% C1**		**Cohen's d**
**Variables**	***M***	***S.D***	***M***	***S.D***	***t (544)***	***p***	***LL***	***UL***	
Machiavellianism	19.10	3.05	19.24	3.17	−0.507	0.612	−0.659	0.388	0.05
Narcissism	18.47	2.57	17.14	3.33	5.273	0.000	0.836	1.83	0.45
Psychopathy	18.06	2.71	14.41	3.54	13.637	0.000	3.12	4.18	1.16
Psychological Maladjustment	98.65	15.22	100.03	15.88	−1.037	0.300	−4.00	1.24	0.09
Psychological Distress	27.12	9.04	29.17	9.17	−2.627	0.009	−3.59	−0.518	0.23
Subjective Happiness	17.48	4.84	18.93	4.27	−3.680	0.000	−2.22	−0.675	0.30

*CI, Confidence Interval; UL, Upper Limit; LL, Lower limit*.

**Table 3:** Descriptive statistics and partial correlation of study variables.

	**Variable**	***n***	***M***	***SD***	**1**	**2**	**3**	**4**	**5**	**6**
1	Machiavellianism	546	19.17	3.11	-					
2	Narcissism	546	17.86	3.01	0.244**	-				
3	Psychopathy	546	16.39	3.61	0.285**	0.295**	-			
4	Psychological Maladjustment	546	99.28	15.5	0.183**	0.120*	0.330**	-		
5	Psychological Distress	546	28.06	9.15	0.187**	0.071	0.238**	0.699***	-	
6	Subjective Happiness	546	18.15	4.64	0.111*	0.046	−0.109*	−0.282**	−0.205**	-

^*^p <0.05, ^**^p <0.01, ^***^p <0.001.

*Correlations between the variables are computed by partializing gender*.

To estimate the effect of psychological maladjustment on Dark Triad traits, psychological distress, and subjective happiness regression analyses were performed. According to the multiple regression analysis (1) dark traits- Machiavellianism and psychopathy (predictors) significantly predicted psychological maladjustment (mediator), psychological distress, and happiness (outcome variables); (2) psychological maladjustment (mediator) significantly predicted outcome variables (see Tables 4, 5).

**Table 4:** Regression coefficients of dark triad traits on psychological maladjustment, psychological distress, and happiness.

	**Psychological maladjustment**						**Psychological distress**						**Subjective happiness**					
					**95% CI**					**95% CI**						**95% CI**	
**Variables**	**B**	**SE**	**ß**	**P**	**LL**	**UL**	**B**	**SE**	**ß**	**p**	**LL**	**UL**	**B**	**SE**	**ß**	**p**	**LL**	**UL**
Machiavellianism	0.651	0.214	0.130	0.003	0.23	1.1	0.496	0.129	0.168	0.000	0.24	0.75	0.230	0.065	0.154	0.000	0.10	0.36
Narcissism	−0.042	0.230	−0.008	0.857	−0.49	0.41	−0.117	0.138	−0.039	0.396	−0.39	0.15	0.086	0.070	0.056	0.218	−0.05	0.22
Psychopathy	1.01	0.192	0.235	0.000	0.63	1.4	0.309	0.116	0.122	0.008	0.08	0.54	−0.293	0.058	−0.228	0.000	−0.41	−0.18
	R = 0.291, R^2^ = 0.085 (F = 16.694***)						R = 0.219, R^2^ = 0.048 (F = 9.086***)						R = 0.239, R^2^ = 0.057 (F = 10.9301***)					

*N = 546, B = unstandardized coefficients; SE B = the standard error for the unstandardized beta; ß = the standardized beta.*

^***^p <0.001.

**Table 5:** Regression coefficients of psychological maladjustment on psychological distress and happiness.

	**Psychological distress**						**Subjective happiness**					
					**95% CI**						**95% CI**	
**Variable**	**B**	**SE B**	**ß**	**P**	**LL**	**UL**	**B**	**SE B**	**ß**	**P**	**LL**	**UL**
Psychological Maladjustment	0.412	0.018	0.699	0.000	0.377	0.448	−0.081	0.012	−0.272	0.000	−0.105	−0.057
	R = 0.699, R^2^ = 0.489 (F = 520.127***)						R = 0.272, R^2^ = 0.074 (F=43.358***)					

*N =546, B = unstandardized coefficients; SE B = the standard error for the unstandardized beta; ß = the standardized beta.*

^***^p <0.001.

Structural equation model depicting significant regression and correlation paths at the level of p <0.05 to *p* < 0.001. The fit indices for the modified model were acceptable: *p* = 0.375, χ2(5) = 5.35, RMSEA = 0.01, GFI = 0.99, TLI = 0.99, CFI = 0.99, NFI = 0.99, IFI = 0.99, χ2/*df* = 1.07.

**Figure 2:**
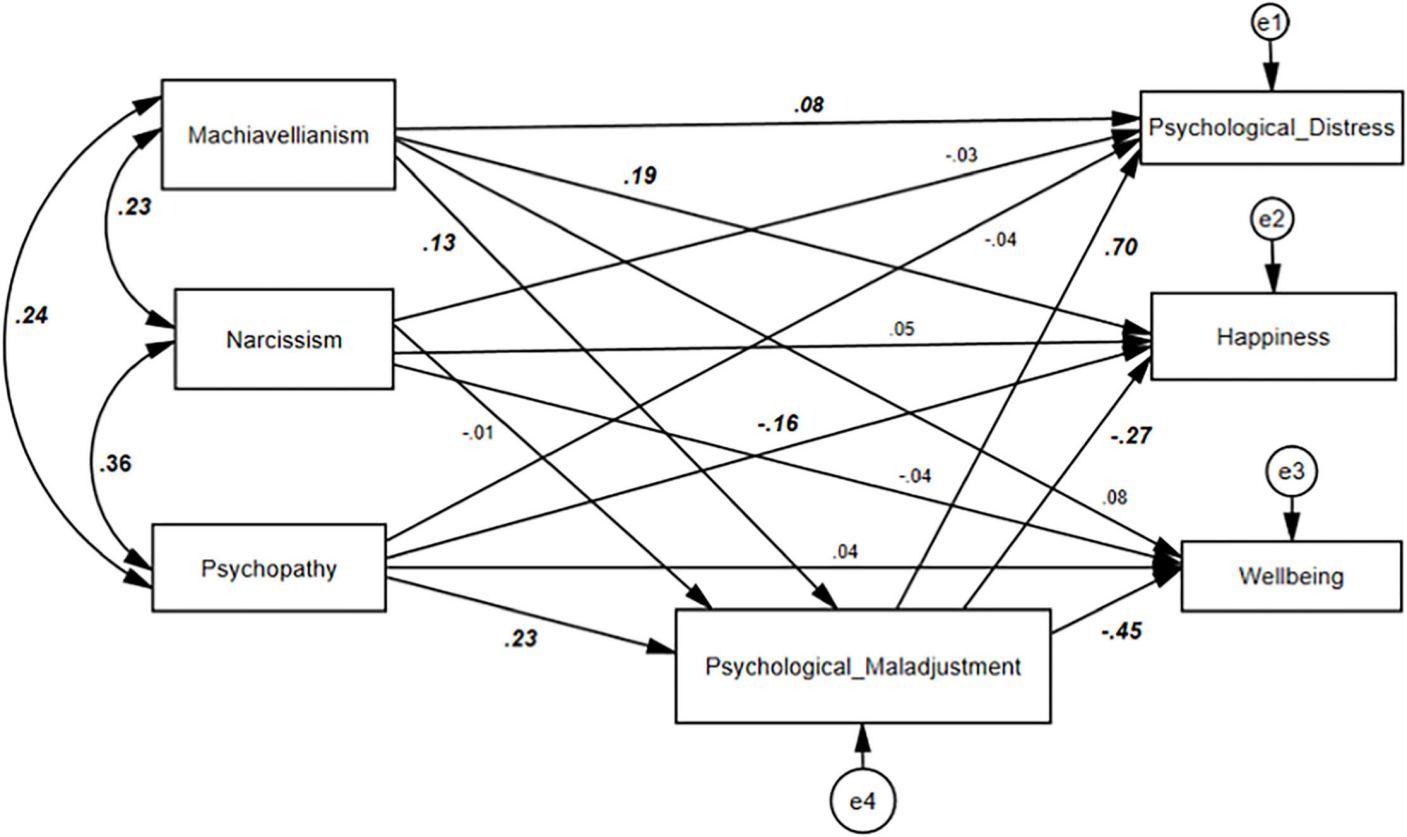
Structure equation model predicting emerging adults' psychological distress and happiness. Significant paths coefficients are presented in bold.

According to the model, psychological distress and subjective happiness were significantly predicted by Machiavellianism and psychological maladjustment, whereas psychopathy significantly predicted subjective happiness with psychological maladjustment. Unstandardized estimates are presented in Table 6; CR > 1.96 for the regression weight represented a significant path at *p* < 0.05. Narcissism was not significantly predicted subjective happiness and psychological distress but was included in the modified model as it was significantly correlated with the other two dark traits (psychopathy and Machiavellianism). Standardized estimates for each path are shown in Figure 2.

**Table 6:** Structural equation model path coefficients.

**Path**	**Estimate**	**S.E**.	**C.R**.	**P**
Psychological maladjustment ← machiavellianism	0.644	0.211	3.056	0.002
Psychological maladjustment ← psychopathy	0.999	0.182	5.504	0.000
Psychological distress ← machiavellianism	0.182	0.092	1.988	0.047
Subjective happiness ← machiavellianism	0.295	0.062	4.737	0.000
Subjective happiness ← psychopathy	−0.189	0.055	−3.467	0.000
Psychological distress ← psychological maladjustment	0.405	0.018	22.157	0.000
Subjective happiness ← psychological maladjustment	−0.080	0.013	−6.415	0.000
Machiavellianism↔ narcissism	2.182	0.411	5.311	0.000
Psychopathy↔ narcissism	3.899	0.494	7.896	0.000
Machiavellianism↔ psychopathy	2.626	0.492	5.342	0.000

*Estimate, unstandardized regression weights; SE, standardized error; CR, critical ratio, p = significance value*.

## Discussion

The present study was conducted with an objective to explore the mediating role of psychological maladjustment in relation to the Dark Triad, psychological distress, and subjective happiness in emerging adults of Pakistan. There is a scarcity of empirical evidence specifically in the context of emerging adults (who are at a transitional stage from adolescence to adulthood) when talking about the DT traits. Wellbeing is an important domain to study with emerging adults as this transitional stage has the additional burden of searching for personal identity to the responsibilities as young adults to establish a stable career and intimate relations. Literature supports the link between DT and depression, anxiety, and perceived stress (Nock et al., 2010; Harrop et al., 2017; Kajonius and Björkman, 2020). Earlier research mostly focused on emotional experiences, stress, anxiety, and depression with DT, therefore the present study focused on wellbeing with special reference to psychological distress (negative domain) and subjective feelings of happiness (positive indicator). There is a unique addition to the literature to explore the role of psychological maladjustment. Psychological maladjustment is an individual's inability to react successfully and in a satisfied manner to the demands of the environment. An emerging adult with a lack of adjustment can behave in an emotionally unresponsive fashion. He/she can be hostile, aggressive, defensive, and manipulative with negative self-adequacy and mental health problems (Khaleque, 2015). These maladaptive behaviors increase the likelihood of distress in emerging adults.

The obtained results confirmed the relationship between DT and psychological distress. It has been revealed that dark traits particularly Machiavellianism and psychopathy are significantly related to the high levels of psychological distress in emerging adults (H1b and H1c are statistically accepted) whereas narcissism appeared to be a non-significant predictor (H1a is not statistically confirmed). The findings of the current study suggest that Machiavellianism may predispose individuals to psychological distress more strongly than the other dark traits. The previous literature heavily supports the association between Dart Triads traits and depression, anxiety, and stress (Krampen et al., 1990; Cale and Lilienfeld, 2002; Stinson et al., 2005; Vazire and Funder, 2006; Jones and Paulhus, 2009; Kennealy et al., 2010; Miller et al., 2010; Noser et al., 2014; Birkás et al., 2016; Muris et al., 2017; Jonason and Davis, 2018). Literature is heavily embodied in the exploration of narcissism in a negative context; e.g., antisocial personality traits, and psychopathology (Paulhus and Williams, 2002). In recent years, researchers tried to explore narcissism with positive domains, such as intrapersonal adjustment, satisfaction with life, and happiness (Egan et al., 2014; Dufner et al., 2019). Narcissists are usually distinguished into two major types; grandiose and vulnerable (Wink, 1991; Miller et al., 2011). Grandiose narcissism has been linked to positive outcomes, such as social confidence, high levels of motivation, positive self-esteem, need for admiration, self-confidence, and control (Campbell and Miller, 2011); therefore this type of personality is widely correlated with psychological adjustment, relationship satisfaction, positive affect, mental toughness which further reduce the levels of depression, anxiety, loneliness, sadness and perceived stress (Sedikides et al., 2004; Ng et al., 2014; Sabouri et al., 2016; Papageorgiou et al., 2019). Vulnerable narcissism is related to low self-esteem, negative affect, withdrawal, sensitivity, and defensiveness (Wink, 1991). The non-significant results may be due to the non-identification of subtypes of narcissism in the SD3 measure. The present research suggested further exploration of these dark traits into deeper subdomains.

Happiness is an aspect of human flourishing. There is considerable research attempting to link the Dark Triad traits to some measure of dispositional happiness (Aghababaei and Błachnio, 2015; Zajenkowski and Czarna, 2015). Literature is equivocal, with negative predictors of positive affect. By keeping these mixed findings in view, subjective experience of happiness is explored with DT traits. The results are somewhat unique in nature. Narcissism appeared to be non-significant whereas Machiavellianism is positively associated with happiness is contrary to the suggested hypothesis H2b, while H2c is statistically accepted as psychopathy is a significant negative predictor of subjective happiness in emerging adults. Individuals displaying higher scores on psychopathy also show less expression of meaning in life and hope (Bartels and Pizarro, 2011; Berg et al., 2013). The non-significant and positive link between narcissism, Machiavellianism, and happiness (H2a and H2b) can also be looked at from other angles. First, the scale of happiness used in this study predominantly measures the subjective experience of happiness which is a global assessment of whether someone is happy or unhappy. Second, Machiavellianism is a personality trait involving manipulativeness and deceit, a cold, calculating attitude toward others which may affect their responses to subjective happiness differently than just feeling “pleased,” “excited,” “annoyed” etc. (Diener, 1994; Lyubomirsky and Lepper, 1999).

Maladjustment is a term usually represented in the form of anger, aggression, threatening, and hostile response system, and a negative view of the world (Rohner, 2004). There is a vast representation of literature on maladjustment or negative aspects of personality in relation to DT traits (Paulhus and Williams, 2002; Jakobwitz and Egan, 2006; Grigoras and Wille, 2017; Muris et al., 2017; Lyons, 2019). In the present study, psychological maladjustment is significantly related to DT when controlling the effects of gender, in the same way, psychological maladjustment is a positive predictor of psychological distress and a significant negative predictor for subjective happiness in emerging adults. When talking about the mediating role of psychological maladjustment, it has been determined that psychological distress and happiness are mediated by this psychological maladjustment only in the case of Machiavellianism and psychopathy (H3a, H3c, H4a, and H4c have statistically confirmed the significant path). The findings of the current research confirmed further that the three distinct subtypes of DT are correlated with each other (see the significant standardized coefficients covariances in Figure 2). This study expands on the previous findings by demonstrating the mediating effect of psychological maladjustment on psychological distress and subjective happiness. The mediating effect of psychological maladjustment on outcome variables (i.e., psychological distress and happiness) indicated that Machiavellianism could directly and indirectly, *via* the impairment of psychological adjustment, exacerbate psychological distress in emerging adults, whereas Machiavellianism and psychopathy decrease subjective happiness in emerging adults. The study result is in line with prior research showing high levels of DT are highly predictive of the negative consequences. The study results also suggest that individuals with high levels of maladjustment are more likely to develop distress and unhappiness (Paulhus and Williams, 2002; Rohner, 2004; Grigoras and Wille, 2017; Lyons, 2019). The connection between DT, psychological distress, and happiness is evidently manifested through the mediation of psychological maladjustment.

There are significant implications of the present study. The results confirm the relationship between DT traits and wellbeing in emerging adults and psychological maladjustment is an important factor that contributes significantly to this relation. The inclusion of adult attachment as a major concern of this developmental stage can explain the deep-down connections between Dark Triad personality traits and relational wellbeing. The practical implication can be the possibility of mitigating the harmful effects of DT personality traits by forming healthy relations and implying the psychological adjustment and related constructs of empathy, and perspective-taking to moderate the association of DT traits and psychological distress.

### Limitations and Suggestion

The following limitations of the study should be noted. *First*, the cross-sectional design was used which limited the ability to draw a causal relationship between the variables. *Moreover*, due to convenience samples of university students, we cannot ignore the sampling biases. *Furthermore*, the study was conducted on emerging adults only, therefore differences in age groups did not emerge, future researchers can conduct studies with adolescents, emerging adults, established adults, and midlife adults to explore DT traits deeply. *Next*, the study data is based solely on self-report measures with online administration that may have several potential biases and can easily produce common method variance. *Finally*, the partial mediation effect of maladjustment may suggest the role of other protective and risk variables (e.g., adverse life events, childhood abuse or trauma, impulsivity, attachment, empathy, emotional intelligence, resilience, etc.).

## Conclusion

The present study assessed the association of Dark Triad traits with psychological distress and happiness among emerging adults. This relationship was further evaluated in the light of psychological maladjustment as a mediator. Overall results of the study demonstrated that the effects of Machiavellianism and psychopathy on psychological distress are exacerbated by psychological maladjustment. The following limitations of the study should be noted. *First*, the cross-sectional design was used which limited the ability to draw a causal relationship between the variables. *Next*, the study data is based solely on self-report measures with online administration that may have several potential biases and can easily produce common method variance. *Finally*, the partial mediation effect of maladjustment may suggest the role of other protective and risk variables (e.g., adverse life events, childhood abuse or trauma, impulsivity, attachment, empathy, emotional intelligence, resilience, etc.).

## Data Availability Statement

The original contributions presented in the study are included in the article/supplementary material, further inquiries can be directed to the corresponding author.

## Ethics Statement

The studies involving human participants were reviewed and approved by Ethical Review Committee National University of Modern Languages, Islamabad, Pakistan. The patients/participants provided their written informed consent to participate in this study.

## Author Contributions

AM and AI designed the study, wrote and revised the manuscript, guided the data entry, and did an analysis. AM collected the data, did part of the data analysis, and wrote part of the manuscript. AA performed the literature review, collected the data, and did the data entry. EE-C did part in data analysis and wrote part of the manuscript along with the review. All authors contributed to the article and approved the submitted version.

## Conflict of Interest

The authors declare that the research was conducted in the absence of any commercial or financial relationships that could be construed as a potential conflict of interest.

## Publisher's Note

All claims expressed in this article are solely those of the authors and do not necessarily represent those of their affiliated organizations, or those of the publisher, the editors and the reviewers. Any product that may be evaluated in this article, or claim that may be made by its manufacturer, is not guaranteed or endorsed by the publisher.
